# Interactions between Tumor Cells, Neurons, and Microglia in the Glioma Microenvironment

**DOI:** 10.3390/ijms21228476

**Published:** 2020-11-11

**Authors:** Daniel P. Radin, Stella E. Tsirka

**Affiliations:** Stony Brook Medical Scientist Training Program, Molecular and Cellular Pharmacology Graduate Program, Department of Pharmacological Sciences, Renaissance School of Medicine, Stony Brook University, Stony Brook, New York, NY 11794-8651, USA; daniel.radin@stonybrookmedicine.edu

**Keywords:** AMPA receptor, CSF1R, glioma, microglia

## Abstract

Despite significant strides made in understanding the pathophysiology of high-grade gliomas over the past two decades, most patients succumb to these neoplasias within two years of diagnosis. Furthermore, there are various co-morbidities associated with glioma and standard of care treatments. Emerging evidence suggests that aberrant glutamate secretion in the glioma microenvironment promotes tumor progression and contributes to the development of co-morbidities, such as cognitive defects, epilepsy, and widespread neurodegeneration. Recent data clearly illustrate that neurons directly synapse onto glioma cells and drive their proliferation and spread via glutamatergic action. Microglia are central nervous system-resident myeloid cells, modulate glioma growth, and possess the capacity to prune synapses and encourage synapse formation. However, current literature has yet to investigate the potential role of microglia in shaping synapse formation between neurons and glioma cells. Herein, we present the literature concerning glutamate’s role in glioma progression, involving hyperexcitability and excitotoxic cell death of peritumoral neurons and stimulation of glioma proliferation and invasion. Furthermore, we discuss instances in which microglia are more likely to sculpt or encourage synapse formation during glioma treatment and propose studies to delineate the role of microglia in synapse formation between neurons and glioma cells. The sex-dependent oncogenic or oncolytic actions of microglia and myeloid cells, in general, are considered in addition to the functional differences between microglia and macrophages in tumor progression. We also put forth tractable methods to safely perturb aberrant glutamatergic action in the tumor microenvironment without significantly increasing the toxicities of the standard of care therapies for glioma therapy.

## 1. Introduction

Gliomas are the most prevalent primary malignant tumor type of the central nervous system (CNS), constituting more than 80% of malignant CNS tumors in the United States [1]. Glioblastoma (GB), the highest grade of glioma, is the most aggressive and common glioma subtype in adults [1]. The standard of care consists of maximal safe surgical resection, followed by fractionated radiation therapy paired with the alkylating agent temozolomide. Recently, tumor-treating field technology consisting of alternating electrical fields to interfere with metaphase and anaphase of rapidly dividing cells was introduced as an additional tool to manage these aggressive neoplasms. However, despite the multiple modalities tailored to treat GB, median survival remains ~20 months with near universal lethality [2]. Further, patients with GB exhibit multiple co-morbidities, including headaches, sleep disturbances, neurological deficits, and pharmacoresistant seizures [1,2,3,4].

Mounting literature demonstrates that gliomas utilize various mechanisms to subvert multiple cell types in the CNS to fuel their growth and disease progression, including neurons [5,6,7], astrocytes [8], oligodendrocyte precursor cells (OPCs) [9], and the myeloid cell populations macrophages and microglia [9,10,11]. Elegant studies point to the possibility that curbing oncogenic interactions between glioma cells and these cell types could slow tumor progression [6,7,10,12,13] and, in some cases, may sensitize gliomas to the standard of care therapies [14,15,16]. Targeting tumor cell-specific interactions with non-malignant cells engulfed in a rapidly expanding tumor or on the border of a tumor has the potential to curb disease progression while producing minimal off-target toxicities.

Recently, a series of sophisticated studies have described the biophysical basis for the pro-tumorigenic interactions between glioma cells and peritumoral neurons [17,18]. These studies have expanded upon our understanding of biophysical interactions between neurons and glioma cells, namely that neurons synapses onto glioma cells and fuel their growth via the activation of α-amino-3-hydroxy-5-methyl-4-isoxazolepropionic acid (AMPA)-type glutamate receptors on glioma cells [17,18]. This de novo synaptic input generates coordinated calcium transients that spread to glioma cells connected via tumor microtubes (TMs) [19] and results in a calcium-dependent increase in glioma invasiveness and growth.

As researchers further study and characterize oncogenic synapses between neurons and glioma cells, and TMs connecting glioma cells, it is important also to consider the possible involvement of cell types, such as microglia, which are principally involved in generating, sculpting, and destroying synapses [20,21]. In this review, we set out to discuss our current understanding of how dysregulated glutamatergic synaptic activity drives glioma progression and what therapies currently exist or could be developed to curb this aberrant behavior. Further, we present literature supporting a role for microglia in the generation, upkeep, and modulation of synapses between neurons and glioma cells and suggest studies that would clearly delineate their role in this phenomenon. We also discuss the sex-dependent actions of microglia specifically, and myeloid cells in general, in promoting or curbing glioma progression and the dissimilar roles of microglia and macrophages in modulating tumor growth. In doing so, we hope to lend credit to the hypothesis that immunomodulation may also prove effective in curbing oncogenic interactions between neurons and glioma cells.

## 2. Neurotransmitter/Neuronal Modulation of Glioma Progression

### 2.1. Pathophysiological Hallmarks of Aberrant Glutamate Secretion

It is well established that seizures are a hallmark co-morbidity of glioma [3,22,23,24]. Available evidence suggests that 30–60% of glioma patients experience seizures during the disease course, with approximately two-thirds of seizures occurring at presentation and the last third occurring during treatment [22,23,24]. Studies conducted over the past twenty years demonstrated that glioma cells secrete significant concentrations of the excitatory neurotransmitter glutamate. Glioma cells are capable of increasing the extracellular glutamate concentration in glutamate-depleted culture medium from 1 μM to ~500 μM in under 48 h [25]. The aberrant glutamate release by glioma cells is primarily mediated by the xCT glutamate/cystine antiporter [25,26,27,28,29,30,31], whose surface expression is regulated by interaction with the epidermal growth factor receptor (EGFR) [30]. Clinical data also exist that support this finding, as Marcus et al. [32] demonstrated using micro dialysis. They found that glutamate levels in glioma tissue surpass 300 μM, while in peritumoral tissue, extracellular glutamate levels are below 10 μM.

The rapid and robust increase in extracellular glutamate was sufficient to induce cell death of neurons co-cultured with the glioma cells via hyperactivation of ionotropic AMPA- and N-methyl-D-aspartate receptor (NMDA)-type glutamate receptors (AMPAR and NMDAR) [25]. As well, autocrine activation of glioma-derived NMDAR and AMPAR resulted in increased glioma invasion [33] and tumor growth, suggesting that pharmacological targeting of relevant glutamate receptors (NMDAR, AMPAR) or systems that mediate glutamate release (EGF, xCT) may slow disease progression while simultaneously limiting common disease co-morbidities. An interesting dichotomy exists in that AMPAR/NMDAR activation on neurons promotes excitotoxic cell death, while activation of the same receptors on glioma cells supports proliferation and invasion. Published literature on glioma-derived AMPAR and NMDAR suggests that glioma cells downregulate these receptors in a grade-dependent manner to support survival in a glutamate-rich environment [34]. This finding is also supported by data in The Cancer Genome Atlas [35]. While AMPAR and NMDAR are downregulated at the transcriptional level, spatial expression analyses support the fact that their expression is highest at the leading edge and in the infiltrating front of the tumors [36], supporting a putative role for them in mediating tumor expansion and invasion into neighboring tissues.

### 2.2. GABAergic Signaling in the Glioma Microenvironment

In parallel to the studies on aberrant glutamatergic signaling in the glioma microenvironment, attention has been paid to dysregulated GABAergic (γ-Aminobutyric acid) signaling and how it may contribute to brain-tumor related epilepsy and glioma progression. Previous reports examining GABAergic signaling in the peritumoral area found dysregulated expression of chloride transporters Na-K-Cl cotransporter 1 (NKCC1) and Chloride potassium symporter 5 (KCC2) specific to neurons [37,38,39]. NKCC1 and KCC2 levels were increased and decreased, respectively, in peritumoral neurons, which resulted in an increase in intracellular chloride concentration from 10 to 15 mM [37]. As a consequence, GABA receptor activation resulted in chloride efflux from peritumoral neurons producing functional excitation [37,38,39]. This functionally depolarizing effect of GABA_A_ receptor activation was further evidenced by the fact that the specific antagonists, picrotoxin and gabazine, ablated epileptic discharges in peritumoral neurons [39].

Mechanistically, these findings beg the question as to why chloride homeostasis is perturbed in peritumoral neurons. Lee et al. [40] carefully delineated the functional interactions between glutamate receptor activation and modulation of the chloride transporter KCC2, principally involved in promoting chloride extrusion from cells. They observed that GABA application, following glutamate exposure, depolarized neurons, and that NMDA receptor antagonism ablated the ability of GABA to produce functional excitation [40]. Glutamate concentrations as low as 20 μM were able to reduce surface and total KCC2 levels, in a manner reversed by co-application of an NMDA receptor antagonist. Translating these findings to glioma, it should be noted that glutamate concentrations in the tumor microenvironment can easily surpass 100 μM [32], and thus robustly downregulate KCC2 levels and shift GABA from an inhibitory to functionally excitatory neurotransmitter.

With specific respect to glioma cells, there exists relatively little literature describing the consequences of GABAergic signaling. Available literature suggests that glioma cells express functional GABA_A_ receptors and that endogenous GABA_A_ receptor activity slows glioma cell proliferation [41]. However, additional exogenous GABA_A_ receptor activation with the specific agonist muscimol did not limit cell proliferation further. Careful analysis detailed that GABA seems to act principally on glioma cells with stem-like features often referred to as glioma stem cells (GSC). These data present GABA signaling in a paradoxical light: On the one hand, GABA signaling seems to stifle glioma cell proliferation and tumor progression while simultaneously serving to functionally excite peritumoral neurons, which may contribute to the genesis of brain tumor-related epilepsy.

### 2.3. Aberrant Communication between Glioma Cells and Peritumoral Neurons

Previous studies have elucidated the pro-proliferative effect neuronal activity has on neuronal and oligodendrocyte precursor cells, the cells principally believed to undergo an oncogenic transformation in the CNS and give rise to various types of high-grade gliomas [42]. Venkatesh et al. [6] characterized oncogenic actions of neuronal activity in various types of high-grade glioma. They established a model in which deep cortical projection neurons expressed channelrhodopsin-2 (ChR2), and an optical fiber inserted below the pial surface could be used to induce action potentials and reported that neuronal stimulation increased glioma cell proliferation without significantly affecting tumor cell death [6].

Proteins between 10 and 100 kDa were found to be responsible for the activity-dependent increase in glioma cell proliferation. Quantitative mass spectrometry implicated neuroligin 3 (NLGN3) as a primary driver of this paracrine growth axis. Brain-derived neurotrophic factor (BDNF) release also accelerated glioma cell proliferation. Mechanistically, NLGN3 can induce Fos expression, activate PI3K, Akt, and mTOR, and further induce NLGN3 expression in glioma cells [6]. In a subsequent study, the authors uncovered that glioma cells derived from pediatric glioma, diffuse intrinsic pontine glioma, and adult glioblastoma did not grow in NLGN3 knockout mice [7]. NLGN3 was also found to stimulate FAK upstream of PI3K/mTOR and increased synapse-related genes in the glioma cells. This finding parallels data showing that specific PIK3CA mutations result in the induction of synaptic gene expression in glioma [43]. Taken together, these findings suggest that glioma-intrinsic and extrinsic factors may regulate synaptogenic action in glioma.

Venkatesh et al. revealed that ADAM10 cleaved NLGN3 from neurons and OPCs and that NLGN3, but not other neuroligins, stimulated various pathways, including FAK-PI3K-Akt-mTOR, Src, and Shc-Ras-Raf-Mek-Erk pathways. This may be explained by the finding that NLGN3 activates integrin Beta 3, EGFR, fibroblast growth factor receptor, and the vascular endothelial growth factor receptor [7]. Importantly, tetrodotoxin (TTX) was able to limit NLGN3 secretion, providing further support for the activity-dependent release of this protein into the tumor microenvironment. Further, pharmacological inhibition of ADAM10 reduced NLGN3 cleavage and slowed growth in vivo.

With evidence that NLGN3 induces expression of various synaptic genes in glioma cells, workers continued to probe the possibility that glioma cells and neurons synaptically engage one another and that this putative interaction could also serve as a hallmark of activity-dependent glioma progression. Venkatesh et al. [18] demonstrated in pre-treatment glioma biopsies that glioma cells express various glutamate receptor subunits and genes associated with post-synaptic structures. Further, enrichment of these genes was found in glioma subpopulations that resembled OPCs. Approximately 10% of glioma cells had developed neuro-glioma synapses (NGS), and that the development of these synapses was reduced in NLGN3 KO mice. They detected fast inward currents that strongly resembled excitatory post-synaptic currents (EPSCs). Such currents could be blocked by the competitive AMPAR antagonist 2,3-dioxo-6-nitro-7-sulfamoyl-benzo[f]quinoxaline (NBQX) and the Ca-permeable AMPAR antagonist 1-napthyl acetyl spermine (NASPM) [18].

A percentage of glioma cells with low input resistance also exhibited sustained currents (>1 s) that were antagonized by TTX but not NASPM. Gap junctions between astrocytes explain low membrane resistance [44]. Glioma cells were shown previously to interconnect via gap junction-coupled TMs [19], so authors reasoned that prolonged currents were spread through these connections. In accordance with this hypothesis, gap junction blockers carbenoxolone and meclofenamate diminished the amplitude of prolonged glioma currents, a finding which further bolsters the theory that glioma cells share physiologically pertinent intercellular connections [18].

AMPAR calcium gating depends critically on the presence or absence and editing state of Glur2 (GluA2, Gria2). Functional AMPAR tetramers without Glur2 or with a Glur2 subunit that has not undergone editing of glutamine to arginine in the second hydrophobic segment are calcium-permeable [45,46]. Venkatesh [18] and others [47,48] reported that the Glur2 subunit is under-edited in glioma, suggesting increased calcium permeability of glioma-derived AMPARs. To interrogate specific contributions of Glur2 to glioma progression, authors overexpressed Wild-type (WT)- and dominant negative (DN)-Glur2 in glioma cells. Not surprisingly, mice bearing tumors overexpressing WT- and DN-Glur2 exhibited reduced and increased survival, respectively. Further, glioma cell proliferation was reduced by treatment with the non-competitive AMPAR antagonist perampanel [18]. In a monoculture, glioma cells overexpressing DN-Glur2 displayed no difference in proliferation or apoptosis but did display reduced invasive capacity. Importantly, the competitive AMPA antagonist NBQX did not reduce glioma proliferation in monoculture but did reduce the increased proliferation observed when glioma cells were co-cultured with neurons, in line with data that perampanel principally reduced glioma cell proliferation in vivo [18]. While these results elegantly define the precise oncogenic roles of Glur2, the oncogenic roles of the other AMPAR subunits Glur1, 3 and 4 still require further study.

A companion paper explored the glutamatergic component of NGS further [17]. Venkataramani et al. documented the presence of multiple NGS on TMs of glioma cells but not in curable meningiomas and oligodendrogliomas [17]. Using super-resolution 3D direct stochastic optical reconstruction microscopy (dSTORM), they identified post-synaptic density markers HOMER1-3 and glutamatergic synaptic vesicle clusters. AMPARs were also present in NGS and mostly expressed in isocitrate dehydrogenase (IDH)-mutated astrocytoma cells. Venkataramani et al. also reported under the editing of Glur2, especially in astrocytomas, compared with oligodendrogliomas characterized by 1p/19q co-deletion [17]. They also found that intratumoral connectivity strongly correlated with the AMPAR subunit Glur1.

To further delineate the AMPAergic nature of NGS, patch–clamp recordings measured spontaneous EPSCs (sEPSCs) in vivo, brain slices of xenografted mice, and glioma cell/neuronal co-culture. The competitive AMPAR antagonist 6-cyano-7-nitroquinoxaline-2,3-dione (CNQX) markedly inhibited sEPSCs, as did NASPM [17]. Venkataramani et al. also observed slower inward currents in co-cultures and brain slices of tumor xenograft-bearing mice. Interestingly, CNQX reduced slower inward currents to a greater extent than did NASPM, reinforcing that calcium-impermeable AMPARs may participate in the generation of slow inward currents. As described in the companion paper [18], slow inward currents were also impeded by gap junction antagonism with meclofenamate [17]. Evidence was also presented that AMPA antagonism reduced the proliferation of glioma cells when co-cultured with neurons and reduced proliferation and invasion in vivo [17].

## 3. Microglia: Potential Role as Synaptic Sculptors?

### 3.1. Context-Dependent Actions on Synapse Formation and Pruning

Microglia are CNS-resident myeloid cells that migrate to the developing brain early in gestation [49]. While their function has been principally studied in the setting of CNS disease, it is now more widely recognized that they play important homeostatic roles in the healthy and developing brain [50]. For example, a study looking at young mice reported that microglia phagocytose retinal ganglion cell presynaptic inputs robustly in P5 mice, a phenomenon that was largely diminished in P9 and P30 mice [21]. Interestingly, phagocytic activity seems to be dependent upon neuronal activity. When one eye of a mouse was treated with vehicle or TTX to reduce neuronal activity, a significant increase in synaptic engulfment in the TTX-treated eye was evident [21]. This may bear some interesting implications in the evolving theory that suggests neuronal activity leads to NLGN3-dependent sustained NGS formation in glioma [18]. These and other authors [21,51,52] also provide data supporting the idea that the complement cascade and, more specifically, the C1q and C3 complement proteins are integral to the microglial phagocytic capacity of presynaptic inputs.

While microglia are responsible for synaptic pruning in both healthy [21,51,52] and pathological states [53], they are also mediators of synapse formation, typically in an activity-dependent manner [20,54]. Microglia may mediate synapse formation by direct contact [55] or through the secretion of soluble proteins, such as BDNF [20] or IL-10 [56]. Interestingly, glioma stem cells induce IL-10 secretion by microglia [57], suggesting the presence of a bidirectional signaling axis in which glioma stem cells induce IL-10 secretion by microglia, which can then lead to aberrant synapse formation, an avenue of future research.

### 3.2. Pilot Studies to Discern the Role of Synapse Formation and Maintenance

To date, the studies published provide substantial evidence that NGS are essential for the proliferation and invasion of glioma [17,18]. Their presence and functionality have been well characterized, and there exists some data on how NGS are established and maintained. However, the current literature has not examined the role microglia may play in the formation, maintenance, or destruction of NGS. To that end, a simple experiment would be to establish glioma xenografts and allow sufficient time for NGS to form. Then, mice should be treated with lipopolysaccharide (LPS), either locally or systemically, to induce robust pro-inflammatory microglial activation. Of note, LPS has previously been shown to induce microglial-mediated synapse loss [58], and since microglia make up a significant portion of glioma [59], treatment with a pro-inflammatory mediator, such as LPS, should quickly reveal whether microglia have the potential to perturb or destroy these aberrant synapses. If promoting pro-inflammatory microglial activation is sufficient to curb oncogenic interaction between neurons and glioma cells, further translational studies with safer drugs, such as the FDA-approved antifungal Amphotericin B, may be worth pursuing [60].

In lieu of pharmacological microglial activation, whole-brain radiation may also be used to induce microglia activation in an effort to promote synaptic pruning [61]. Unfortunately, it also appears that microglia are responsible for radiation-induced cognitive deficits and that elimination of microglia or CSF1R blockade reduces cognitive impairment and boosts radiation efficacy [14,16,61,62,63]. Thus, while radiation may be used as a tool to study the effect of microglia on NGS, radiation will most likely not produce robust synaptic pruning once this treatment is paired with CSF1R antagonists in a clinical setting.

## 4. Sex-Specific Microglial Modulation of Glioma Progression

Efforts to globally uncover the role of microglia in neurological diseases have demonstrated specific sex-dependent microglial physiology/pathophysiology (reviewed by [64]). The response of microglia to the type of stress/neurological insult seems vastly dependent upon multiple factors, including age [64]. Further, what seems to stand out is that microglial sex underlies the morphological alterations and expression changes in multiple immunomodulatory proteins in response to neurological stress or disease induction. For example, prior work has demonstrated that female microglia residing in the prefrontal cortex are at an increased state of activation at baseline but that acute and chronic stress reduces microglial cell activation. Conversely, acute and chronic stress does not reduce male microglial cell activation [65]. Further, it seems that estrogen downregulates cortical expression of C-C motif chemokine ligand 2 (CCL2) and transforming growth factor-beta 1(TGF-B1) [66]. TGF-B1 is markedly important for glioma invasion and stemness, while CCL2 acts as a potent chemokine for blood derived monocytes and T-regulatory cells [67].

In the context of glioma, there has been little literature published focusing on discriminating the behavior of male and female microglia in the tumor microenvironment. However, recent work demonstrates that junctional adhesion molecule A (JAM-A) may function as a female microglial tumor suppressor. In their studies, Turaga et al. demonstrated that female mice live longer after intracranial injection of syngeneic GL261 murine glioma cells [68]. When JAM-A is deleted in male and female mice, male mice exhibited a slight survival advantage. Knocking out JAM-A did not significantly alter male survival but significantly reduced female mouse survival [68]. Further interrogation into the oncogenic phenotype revealed that microglia are more activated in JAM-A deficient females and that the expression of anti-inflammatory genes in female microglia increases when JAM-A is knocked out. In line with a putative anti-inflammatory phenotype, JAM-A deficient female microglia are more phagocytic and enhance the proliferation of GL261 glioma cells in vitro [68]. These data also suggest that while anti-inflammatory microglia can suppress anti-cancer immune defenses, they may also release factors to drive glioma proliferation directly. Such results may begin to explain why women with glioblastoma exhibit a marginally increased survival over men [69,70].

Pediatric patients with Neurofibromatosis type 1 (NF1) are at an increased risk of developing lower-grade tumors on the optic nerve. A significant subset of these patients experiences visual impairment during and after treatment. Of particular interest, female patients are significantly more likely to lose their vision. This finding was interrogated previously by Toonen et al. [71]. Using an NF1 optic glioma mouse model, they determined that female optic gliomas harbor a 3-fold increased level of microglia compared to male optic gliomas. Further, estrogen receptor B antagonism minimized retinal ganglion cell loss, implicating estrogen as a key mediator of clinical co-morbidities of this disease [71]. There also exist additional pre-clinical and clinical data regarding the sex-dependent properties of myeloid cells in glioma progression. While not specific to microglia, it has been observed that proliferating monocytic myeloid-derived suppressor cells are predominant in tumors in males and that granulocytic myeloid-derived suppressor cells are enriched for in the tumors of female patients. Further, IL1B exhibits prognostic utility in females but not in males [72]. Taken together, these data suggest that immunotherapies for glioma patients may also need to be tailored to the sex of a particular patient.

## 5. Microglia and Macrophages: Partners in Crime or Divergent Players in Glioma?

There now exists mounting evidence that demonstrates the crucial role microglia and macrophages play in multiple aspects of glioma progression. However, to date, there exists relatively little literature that attempts to separate out the specific roles each myeloid cell type plays in the progression of glioma. Available evidence suggests that microglia are more pro-inflammatory in IDH-mutant gliomas, which may contribute to increased survival times [73]. Additional investigations of IDH-mutant tumors illustrate an increase in macrophage transcriptional programs over microglia with increasing tumor grade [74]. In IDH-mutant gliomas, microglia comprise the majority of the infiltrating immune cells, which may be explained by their increased proliferative nature compared to macrophages in this glioma subtype [75].

There is a paucity of evidence regarding the disparate functional roles of microglia and macrophages in glioma. However, we can infer function from their spatial distribution in the tumor. Our lab undertook significant efforts to integrate preclinical and clinical data to extrapolate specific functions of macrophages and microglia in the glioma microenvironment. Using available clinical and preclinical data in mouse models of glioma, microglia were found to principally localize to the edge of gliomas where they may facilitate spatially relevant behaviors, such as invasion, proliferation, and stemness [76,77,78,79,80,81,82]. Our lab showed enrichment of peripheral glioma-associated microglia correlates with decreased patient survival [76]. Interestingly, microglia also facilitate the recruitment of anti-inflammatory macrophages and T-regulatory cells from systemic circulation [67,76] by the release of chemokines, including CCL2. Microglia can also promote stemness via the release of IL-6 [81,82], which may, in turn, induce glioma stem cells to recruit anti-inflammatory macrophages via periostin release [83]. Once entrenched in the glioma microenvironment, these tumor-educated macrophages would support angiogenesis [12,13,75,84,85], a finding strengthened by the fact that they form physical contacts with blood vessels to a greater degree than microglia [75]. Additional work has revealed that the macrophage proportion increases in recurrent gliomas, potentially suggesting a macrophage functional role in mediating glioma recurrence after radiation [14]. While considerable work still needs to be done to uncover additional roles of these myeloid cells and to pave the way for tumor-specific therapy, mounting evidence seems to suggest specific and distinct roles for microglia and macrophages dependent on their localization within and around a tumor.

## 6. Translational Considerations

### 6.1. Pharmacological Targeting of Aberrant Glutamatergic Action in Glioma

**Figure 1:**
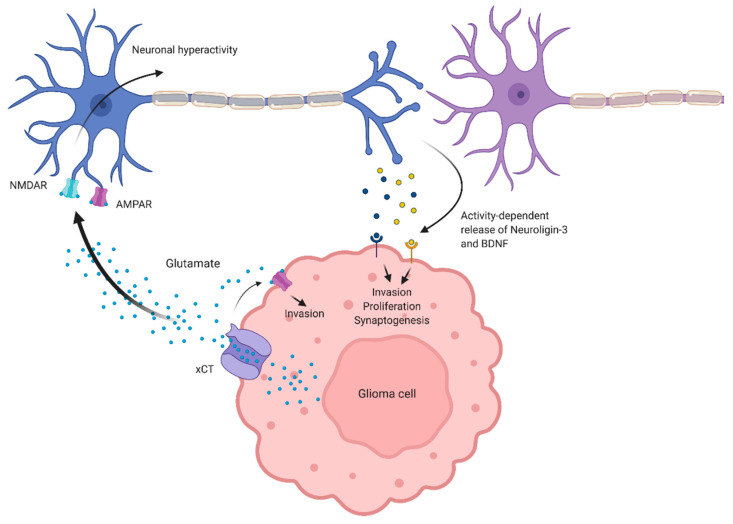
Effects of aberrant glutamate levels in the tumor microenvironment. Glioma-derived glutamate activates α-amino-3-hydroxy-5-methyl-4-isoxazolepropionic acid receptors (AMPARs) on glioma cells to drive invasion. Glutamate also induces cortical hyperexcitability, presumably via activation of AMPARs and N-methyl-D-aspartate receptors (NMDARs) on peritumoral neurons. Neuronal hyperactivity mediates the release of presynaptic proteins, such as brain-derived neurotrophic factor (BDNF) and Neuroligin-3, which drive glioma proliferation, invasion, and synaptogenesis between glioma cells and peritumoral neurons.

Studies published over the last two decades point to several druggable processes to selectively limit glioma growth and progression. Initial studies suggested that glioma cells induced hyperexcitability in neighboring neurons to promote cell death, which would make it easier for glioma cells to invade nearby CNS parenchyma [25,28]. Subsequent investigations revealed that the induction of neuronal hyperactivity resulted in the release of secreted factors by neurons, such as BDNF or NLGN3 (Figure 1), which support glioma progression and facilitate the synapsing of neurons onto glioma cells [6,7,17,18]. With these observations in mind, two possible targets are revealed: the xCT antiporter [86,87] and AMPARs [17,18,25,28,33].

Glioma cell-derived xCT is, at least, partly responsible for promoting cortical hyperexcitability, brain edema, and neuronal cell death [25,86,87,88]. Further, pharmacological antagonism with the FDA-approved compound sulfasalazine diminishes cortical hyperexcitability and augments temozolomide efficacy against glioma cells in vitro [86,89]. Unfortunately, available clinical evidence suggests that sulfasalazine also augments the hematological side effects of radiation + temozolomide [90]. Whether other xCT inhibitors, such as sorafenib or erastin, also enhance temozolomide off-target toxicity remains to be seen [91]. Regardless, pharmacological inhibitors of xCT, such as sulfasalazine, remain useful tools to study its function.

AMPARs also emerge as a promising target to abate multiple oncogenic actions of high-grade gliomas. Antagonizing AMPAR expressed in peritumoral neurons may functionally mimic the effects of xCT antagonism. AMPAR antagonism spares neurons from excitotoxic levels of glutamate secreted from glioma cells [25], which has also been shown to slow glioma growth [28] and reduce cortical hyperexcitability in an in vivo explant model. Whether AMPAR antagonism limits the secretion of mitogenic proteins, such as NLGN3 and BDNF, from neurons has yet to be addressed. Antagonism of glioma-derived AMPARs has also been shown to mitigate the mitogenic effects of neurons synapsing onto glioma cells, supporting the idea that AMPAR antagonism may yield multiple benefits, including in the clinical setting. A phase 2 study was conducted with the non-competitive AMPAR antagonist talampanel on newly diagnosed glioma patients in which talampanel was added to the standard of care regimen. Though the study only included one treatment arm, median survival time of these patient was 20.6 months, with 41.6% of patients surviving 2 year after diagnosis [92]. This is compared to the historical median survival of 14.6 months with 26% of patients surviving 2 years after diagnosis. It is also encouraging that talampanel did not increase the hematological side effects of temozolomide and radiation, suggesting AMPAR antagonists can be safely paired with standard of care modalities [92].

More recently, clinical evidence has emerged suggesting the FDA-approved anti-epileptic AMPAR antagonist perampanel may be useful in curbing glioma growth [93] or at least in controlling glioma-associated epilepsy [94]. In a small clinical study enrolling ten patients, Izumoto et al. administered perampanel up to 8mg per day and reported seizure control in 100% of patients and seizure freedom in 60% of patients [93]. They also reported lesion reduction proportional to the plasma concentration of perampanel. Though causal relations cannot be extrapolated from this small, single-arm study, such data warrant consideration of a larger placebo-controlled trial in newly diagnosed glioma patients. As well, a larger study enrolling 36 patients reported a responder rate of ~90%, with ~33% of patients achieving seizure freedom [94]. Whether additional clinical metrics, such as overall survival rate or progression-free survival rate, are affected by AMPAR antagonism has yet to be conclusively determined, but these initial reports suggest that further studies are worth pursuing.

It is also pertinent to discuss the prospects of modulating other neurotransmitter systems in glioma. NMDAR antagonism may prove to be useful in the management of glioma. Previous studies indicate that NMDAR antagonism, more so than AMPAR antagonism, spares neurons from excitotoxic levels of glutamate secreted by glioma cells [25] and accordingly may reduce peritumoral hyperexcitability. However, previous studies utilizing tumor border tissue resected from glioma patients show that acute AMPAR, not NMDAR antagonism, diminishes cortical GABAergic excitation [39]. Perhaps long-term NMDAR antagonism might restore KCC2 levels in peritumoral neurons [40], which would change GABA from a functionally excitatory back to an inhibitory neurotransmitter in the glioma microenvironment. Additionally, NGS described previously were shown to be primarily AMPAergic. Thus, NMDA antagonism may not be useful in perturbing the direct synaptic interaction between neurons and glioma cells [17]. Nonetheless, the NDMA receptor antagonism directly suppresses the proliferation of glioma cells [95,96] and was well tolerated in glioma patients [97].

As discussed above, GABA action in the glioma microenvironment is markedly paradoxical. Though GABA is thought of as the principal inhibitory neurotransmitter in the mammalian CNS, alterations in glutamatergic signaling in peritumoral neurons convert GABA from an inhibitory to functionally excitatory neurotransmitter [37,38,39]. Available data indicate that augmenting GABA activity in glioma cells does not further reduce proliferation over basal, endogenous activity [41]. Further, GABA acting as an excitatory neurotransmitter may bolster the oncogenic effects of neuronal hyperactivity detailed previously [6]. Therefore, until methods are developed that reliably prevent GABA’s excitatory potential, drugging systems, such as AMPA, may be more clinically useful.

### 6.2. Targeting Myeloid Cells during Glioma Treatment

In addition to targeting aberrant glutamatergic and GABAergic activity in the glioma microenvironment, recent evidence supports the potential clinical utility of targeting myeloid cells to block tumor progression and mitigate the cognitive effects of radiation treatment [10,11,12,13,62,63]. CSF1R blockade on myeloid cells repolarized them and diminished their tumor-promoting activity in vivo [10], though this strategy failed to yield significant clinical benefits in the setting of recurrent glioma [98]. CSF1R antagonism may augment radiation efficacy in the pre-clinical [14,16] and clinical setting [99].

It will be important to design methods by which the onset of cognitive deficits associated with treatments, such as radiation, could be reversed or prevented. As sustained AMPA signaling is vital for cognition and memory [100,101,102,103,104,105,106,107], pairing treatments, such as radiation with perampanel, may prove efficacious in extending patient survival but may also synergistically reduce cognition and promote more significant memory dysfunction in long-term glioma survivors. Fortunately, several groups have reported that targeting microglia abate the cognitive deficits associated with radiation in pre-clinical models, while also extending the survival of glioma-bearing mice [61,62,63].

The studies examining these outcomes typically utilize CSF1R antagonists before radiation treatment. It would be interesting to examine if meaningful CSF1R antagonism could be achieved after exposure to radiation to test whether microglia have the ability to prune NGS, especially given their high abundance in glioma tissue [13,59]. If microglia receive a stimulus, such as ionizing radiation, to prune NGS, immediately followed by CSF1R antagonism, it is conceivable that microglia will utilize their synaptic pruning capabilities to stifle glioma progression while mitigating their long-term deleterious effects on cognition.

While there is a marked and justified focus on antagonizing vital receptors on myeloid cells, such as CSF1R, there is substantial reason to believe that myeloid cells, especially microglia, would functionally respond to pharmacological modulation of both GABA and glutamate receptors. Prior work has shown that microglia express GABAB receptors in addition to several different functional AMPAR subunits [108,109,110,111,112,113]. It has been shown that glutamate induces directed microglial chemotaxis in an AMPAR-dependent manner [114]. Though more research is required to fully parcel out the functional effects of microglial AMPAR activation or antagonism in the setting of glioma, available data suggest that simply by attracting microglia to the edge of the tumor, microglia can be subverted to release ligands that encourage glioma invasion and stemness [9,77,78,79,80,82].

## 7. Conclusions

Mounting evidence supports a role for glutamate in the progression of glioma and for the genesis of multiple co-morbidities that are difficult to manage in the clinical setting. Hyperexcitability and excitotoxicity of peritumoral neurons coupled with the activation of glutamate on glioma cells emerge as critical processes for the growth and spread of glioma cells. More recent research elegantly details glutamatergic NGS as future targets for glioma treatment. Fortunately, blocking AMPARs in the glioma microenvironment appears effective in both pre-clinical and clinical settings, while blocking xCT is a valuable research tool but seems translationally futile.

There are also accumulating data suggesting that microglia can prune synapses or promote their formation depending upon their particular stimulus. Cytokines released from glioma cells, such as IL-10, have the potential to encourage synapse forming activities in microglia, yet this phenomenon has yet to be clearly defined in pre-clinical models. Whether microglia have the capability to modify NGS also has yet to be elucidated, but utilizing pro-inflammatory stimuli, such as LPS or ionizing radiation, can provide a clear answer to this question. It is becoming clear that targeting unique cell surface receptors on microglia, such as CSF1R, can perturb their tumorigenic activities and their deleterious effects on cognition. Ultimately, targeting both oncogenic activity of neurons and microglia in the glioma microenvironment can help manage the co-morbidities and slow the growth of intractable high-grade gliomas.

## Abbreviations

AMPAR	α-amino-3-hydroxy-5-methyl-4-isoxazolepropionic acid receptor
CSF1R	Colony Stimulating Factor 1 receptor
CNS	Central Nervous System
EGFR	Epidermal growth factor receptor
NMDAR	N-methyl-D-aspartate receptor
GABA	γ-Aminobutyric acid
TTX	Tetrodotoxin
NKCC1	Na-K-Cl cotransporter
KCC2	Chloride potassium symporter 5
BDNF	Brain-derived neurotrophic factor
NBQX	2,3-dioxo-6-nitro-7-sulfamoyl-benzo[f]quinoxaline
NASPM	1-napthyl acetyl spermine
WT	Wild-type
DN	Dominant-Negative
IDH	Isocitrate dehydrogenase
CNQX	6-cyano-7-nitroquinoxaline-2,3-dione
LPS	Lipopolysaccharide
CCL2	of C-C motif chemokine ligand 2
TGF-B1	Transforming growth factor – beta 1
JAM-A	Junctional adhesion molecule A
NGS	neuro-glioma synapses
GB	Glioblastoma
OPC	Oligodendrocyte precursor cell
TM	Tumor microtubes
